# Refined pharmacovigilance assessment of immune checkpoint inhibitors-related bullous pemphigoid: a multi-methodological approach utilizing FAERS database

**DOI:** 10.3389/jpps.2025.15597

**Published:** 2026-01-07

**Authors:** Yan Wang, Liu-Yi-Yi Yang, Ya-Gang Zuo

**Affiliations:** Department of Dermatology, State Key Laboratory of Complex Severe and Rare Diseases, PUMC Hospital, CAMS and PUMC, National Clinical Research Center for Dermatologic and Immunologic Diseases, Beijing, China

**Keywords:** adverse drug events, immune checkpoint inhibitors, bullous pemphigoid, pharmacovigilance, FAERS

## Abstract

**Objectives:**

To evaluate the potential risk of bullous pemphigoid (BP) in patients treated with immune checkpoint inhibitors (ICIs) and to characterize ICI-related BP (irBP) using the United States Food and Drug Administration Adverse Event Reporting System (FAERS) database.

**Methods:**

The present study conducted a disproportionality analysis leveraging FAERS database, spanning the first quarter (Q1) of 2004–2025 Q1. To ensure robust signal detection, we employed a quadruple analytical approach incorporating: (1) reporting odds ratio (ROR), (2) proportional reporting ratio, (3) Bayesian confidence propagation neural network, and (4) multi-item gamma Poisson shrinker algorithms. These methodologies were systematically applied to assess the potential risk of BP in patients treated with ICIs. Furthermore, temporal characteristics of adverse event emergence were quantitatively assessed to delineate the time-to-onset patterns.

**Results:**

There are 850 irBP cases identified, comprising reports associated with the following agents: nivolumab (n = 530), pembrolizumab (n = 180), ipilimumab (n = 44), atezolizumab (n = 40), cemiplimab (n = 24), durvalumab (n = 19), tislelizumab (n = 10), and avelumab (n = 3). Affected patients were predominantly males (67.8%) and over 60 years of age (70.1%). All eight ICIs showed positive disproportionality signals, with ROR values ranked descendingly as: cemiplimab > nivolumab > tislelizumab > pembrolizumab > ipilimumab > durvalumab > atezolizumab > avelumab. The median time of irBP onset was 165.2 (IQR: 56–410) days.

**Conclusion:**

The study establishes a significant link between ICIs and BP. All ICIs increase BP risk. CTLA-4 inhibitors exhibited the most marked early risk concentration, highlighting the importance of early dermatologic evaluation after initiating CTLA-4 blockade.

## Introduction

Recently, immune checkpoint inhibitors (ICIs) have been developed as novel therapeutic agents for malignancies, achieving significant anti-tumor responses and extending survival in patients with certain tumor groups [1]. ICIs encompass monoclonal antibodies that target programmed cell death-1 (PD-1), programmed cell death ligand 1 (PD-L1), and cytotoxic T-lymphocyte-associated antigen 4 (CTLA-4). Their anti-cancer effect is mediated by selectively blocking these key immune regulatory pathways, thereby releasing T cell to recognize and destroy tumor antigens [1]. However, this enhancement of anti-tumor immunity can paradoxically lead to nonspecific immune system activation, resulting in a group of toxicities collectively termed immune-related adverse events (irAEs) [2].

Cutaneous irAEs (cirAEs) are the most common irAEs, with a reported incidence approaching 30% in patients treated with ICIs [3]. While the most common cirAEs include nonspecific rash or pruritus, diseases such as eczema, psoriasis and vitiligo are also observed [4]. The mechanism of cirAEs may include epitope spreading and altered T cell subsets [5–7]. Although emerging evidence suggests that cirAEs are associated with enhanced anti-tumor response and improved patient survival outcomes in patients receiving ICIs [8]. CirAEs frequently compromise patients’ quality of life and potentially necessitate discontinuation of ICIs therapy. Bullous pemphigoid (BP) is a subepidermal autoimmune blistering disease and it may also occur from ICIs therapy (ICI-related BP, irBP). ICIs targeting the PD-L1/PD-1 axis can elicit BP in about 0.3%–0.6% patients [9]. In a cohort study of 5636 patients treated with ICIs, 35 (0.6%) developed BP [10]. Notably, irBP patients exhibits distinct clinical features compared to classical BP, such as a prolonged pruritic prodromal phases and extended corticosteroids treatment requirements [3]. Current understanding of irBP remains limited due to small sample sizes in existing studies, and the low prevalence of this condition continues to pose significant challenges in comprehensive clinical characterization.

The US Food and Drug Administration Adverse Event Reporting System (FAERS) is a publicly available database that aggregates voluntary reports of drug-associated AEs from health-care professionals and patients globally. Existing studies on irBP demonstrates notable limitations: (1) The work by Aggarwal et al. [11] while establishing FAERS as a viable data source, was constrained to PD-1 inhibitors (pembrolizumab and nivolumab), with modest case numbers (n = 118). (2) Tan et al. ’s comprehensive FAERS-based study (2011 Q1–2024 Q1), despite employing reporting odds ratio (ROR) methodology across 13-year data, exhibited three key constraints: (a) exclusive reliance on a single disproportionality analysis without complementary method, (b) lack of intra-class agent differentiation, (3) absence of temporal risk quantification.

This study provides a comprehensive pharmacovigilance analysis of irBP by leveraging the FAERS database over an extended period (Q1 2004–Q1 2025). We employed a multi-methodological approach for both signal detection and temporal risk assessment, which included the reporting odds ratio (ROR), proportional reporting ratio (PRR), Bayesian confidence propagation neural network (BCPNN), and multi-item gamma Poisson shrinker (MGPS). By integrating four complementary disproportionality algorithms, we enhanced the robustness and reliability of signal identification. Moreover, we integrated Kaplan–Meier analysis with Weibull shape parameter (WSP) modeling to quantitatively delineate temporal risk patterns. A key advancement in our study was the extension of evaluation beyond the ICI class level to encompass individual agent-level analyses, allowing direct comparisons of clinical characteristics and signal strengths among agents within the same class. Notably, disproportionality analyses consistently showed that PD-1 inhibitors exhibited a higher ROR for irBP compared to CTLA-4 inhibitors, which in turn showed higher ROR values than PD-L1 inhibitors. Collectively, these methodological refinements significantly enhance the depth and breadth of data analysis, providing a solid evidence base for more precise identification and understanding of irBP risk. This, in turn, facilitates the optimization of clinical monitoring and preventive strategies.

## Methods

### Data mining

This retrospective disproportionality analysis utilized FAERS database, accessed from^1^. The study period spanned from the first quarter (Q1) of 2004 to Q1 of 2025. As the study involved analysis of publicly available, anonymized secondary data, it did not require institutional review board approval or direct involvement of human subjects.

The FAERS database includes seven core datasets: demographics (DEMO), drug (DRUG), adverse events (REAC), outcomes (OUTC), report source (RPSR), therapy date (THER), and drug indications (INDI). Reports were included if they listed an ICIs as the primary suspected drug (role_cod = PS). The included ICIs was:

1. PD-1 inhibitors: nivolumab, pembrolizumab, cemiplimab, dostarlimab, tislelizumab
2. PD-L1 inhibitors: atezolizumab, avelumab, durvalumab
3. CTLA-4 inhibitors: ipilimumab, tremelimumab.

Therapy regimens were defined as:

ICI monotherapy: Sole use of one ICI designated as the primary suspected drug.

ICIs combination therapy: Concurrent use of two or more ICIs, with at least one designated as primary suspected drug.

AEs of interest were defined by the MedDRA preferred terms categorized under the standardized MedDRA query for “pemphigoid.”

**FIGURE 1:**
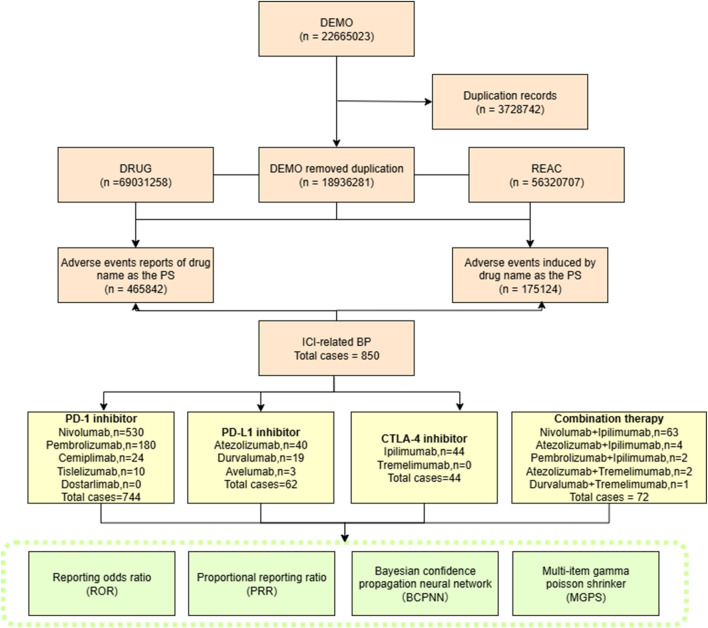
Flow chart showing the selection process of irBP in the FAERS database.

Duplicate reports were removed following FDA’s official guidance: (1) for reports with the same CASEID, only the record with the latest FDA_DT was retained; (2) if both CASEID and FDA_DT were identical, the record with the highest PRIMARYID was included. Subsequently, data of clinical characteristics were collected: gender, age, indications, outcomes, reporters and report countries. A flow diagram of the process is shown in Figure 1.

### Statistical analysis

To evaluate the association between ICIs and BP, four complementary signal detection methods are employed: (1) ROR; (2) PRR; (3) BCPNN: measured via information component (IC); (4) MGPS: estimated via empirical Bayes geometric mean (EBGM). Each method compared the frequency of BP reports with ICI exposure to other AE reports in the FAERS database. Positive signals were defined based on established criteria for each method: (1) ROR >1 with a lower 95% confidence interval (CI) >1 and at least three reports (a ≥ 3); (2) PRR ≥2 with a chi-squared (χ^2^) statistic ≥ 4 and a ≥ 3; (3) IC_025_ >0 for BCPNN; and (4) EBGM_05_ >2 for MGPS (Supplementary Table S1).

Time-to-onset (TTO) was defined as the temporal span between the commencement of ICIs and the onset of BP. To uphold the precision, records featuring erroneous date entries, discrepancies, and omissions were ruled out. TTO was analyzed using descriptive statistics and modeled using the WSP to characterize hazard patterns over time. The Kaplan-Meier method was also utilized to evaluate TTO.

All statistical analyses were conducted using R software (version 4.3.2^2^), and data visualizations were performed using Python (version 3.12). A two-sided *p* value of less than 0.05 was considered statistically significant.

## Results

### Descriptive characteristics: pemphigoid

**FIGURE 2:**
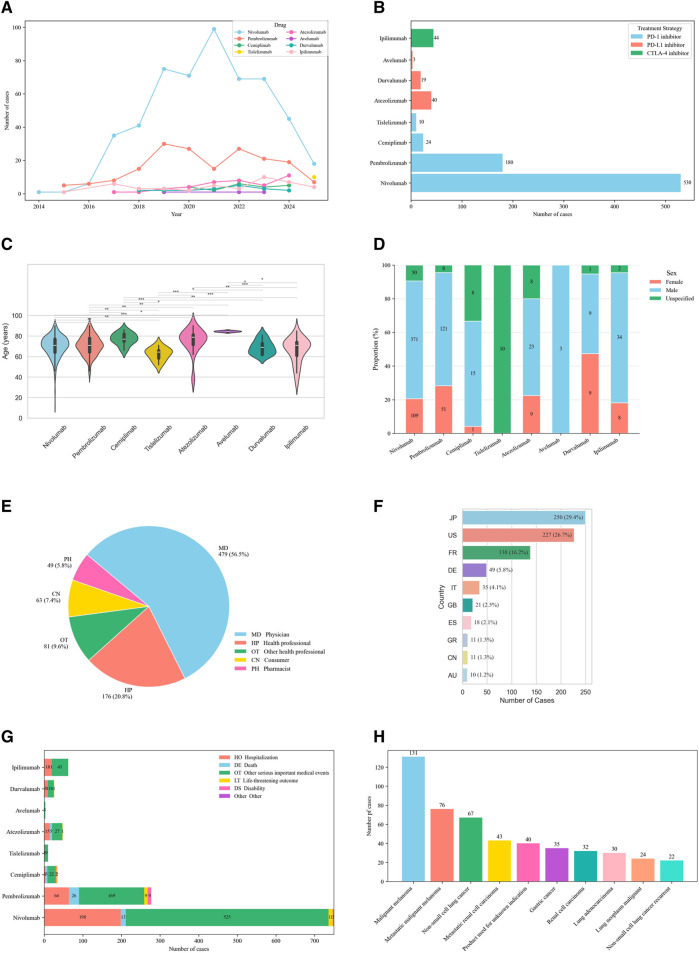
Demographic characteristics of irBP from the FAERS database (Q1 2004–Q1 2025). **(A)** Distribution of reported irBP by years. **(B)** Distribution of cases number by treatment strategy. **(C)** Distribution of patient’s age. Statistical tests were conducted using the Wilcoxon signed rank test. (**p < 0.01,***p < 0.001,****p < 0.0001). **(D)** Distribution of patient’s gender. **(E)** Distribution of reporters. **(F)** Distribution of cases reported by the top ten countries. **(G)** Distribution of patients’ outcome. **(H)** Distribution of cancer types among patients.

Within the FAERS database, 850 irBP cases were identified, in which 744 cases (87.5%) were induced by PD-1 inhibitors, 62 (7.3%) by PD-L1 inhibitors, and 44 (5.2%) by CTLA-4 inhibitors. Seventy-two cases were induced by ICIs combination therapy. The clinical characteristics were detailed in Table 1; Figure 2.

**TABLE 1:** Clinical characteristics of ICIs-BP from the FAERS database (Q1 2004–Q1 2025).

Characteristics	All ICIs	PD-1i	PD-L1i	CTLA-4i
Gender				
Male	576	507	35	34
Female	187	161	18	8
Unspecified	87	76	9	2
Age (years)				
Median	71	71	76	71
<18	1	1	0	0
18–60	105	94	2	9
>60	596	523	45	28
Missing	148	126	15	7
Top 3 reported countries				
​	JP 250	US 209	JP 16	JP 26
​	US 227	JP 208	US 13	FR 8
​	FR 138	FR 119	FR11	US 5
Reporter’s occupation				
Healthcare professional	736	680	62	43
Non-healthcare professional	112	62	-	1
Missing	2	2	-	-
Top 5 indication				
MM (209)	MM (191)	HC (12)		MM (18)
NSCLC (67)	NSCLC (61)	SCLC (5)		RCC (8)
Metastatic RCC (43)	Metastatic RCC (38)	NSCLC (5)		Pleural mesothelioma malignant (3)
Unknown (40)	Unknown (37)	SCC (5)		NSCLC recurrent (2)
GC (35)	GC (35)	Bladder transitional cell carcinoma (4)		Unknown (3)
Outcome				
Hospitalization	307	266	23	18
Life-threatening	24	222	2	-
Disability	14	14	-	-
Missing	1	1	15	-
Death	51	44	6	1
Other	816	727	16	25

CTLA-4i, CTLA-4, inhibitor; PD-L1i, PD-L1, inhibitor; PD-1i, PD-1, inhibitor; JP, japan; US, the United States; FR, France; MM, malignant melanoma; NSCLC, Non-small cell lung cancer; HC, hepatocellular carcinoma; SCC, small cell lung cancer; RC, renal cell carcinoma; SCC, squamous cell carcinoma; GC, gastric cancer.

The cohort was predominantly males (576 cases, 67.8%) versus females (187 cases, 22.0%), with sex unspecified in 87 cases (10.2%). Median patient age was 71 years, with most cases occurring in patients >60 years (596, 70.1%) compared to 18–60 year-olds (105, 12.4%). Geographically, Japan reported the highest number of cases (250, 29.4%), followed by the United States (227, 26.7%) and France (138, 16.2%). Reports originated primarily healthcare professional (736, 86.6%) versus non-healthcare professional (112, 13.2%).

Among 850 irBP cases, most occurred in patients treated for skin and melanoma-related malignancies (260 cases, 30.6%; mainly malignant melanoma, 131 cases), followed by lung cancers (192, 22.6%; mainly non-small cell lung cancer, 67 cases, and lung adenocarcinoma, 30 cases), renal and urinary tract tumors (149, 17.5%; including metastatic renal cell carcinoma, 43 cases, renal cell carcinoma, 32 cases, and bladder/urinary tract tumors, 27 cases), gastrointestinal malignancies (51, 6.0%; mainly gastric and esophageal cancer), head and neck cancers (33, 3.9%), liver malignancies (20, 2.4%), and other or unclassified indications (109, 12.8%). Regarding outcomes, hospitalization was most common (307, 36.1%), followed by life-threatening events (24, 2.8%) and disability (14, 1.65%).

### Disproportionality analysis (signal detection)

Significant pharmacovigilance signals for BP were detected across all eight ICIs analyzed. Significant associations were confirmed for each ICI class:

PD-1 inhibitor (ROR = 22.66, 95% CI 20.99–24.47)
CTLA-4 inhibitor (ROR = 8.79, 95% CI 6.53–11.83)
PD-L1 inhibitor (ROR = 6.55, 95% CI 5.10–8.41)

**FIGURE 3:**
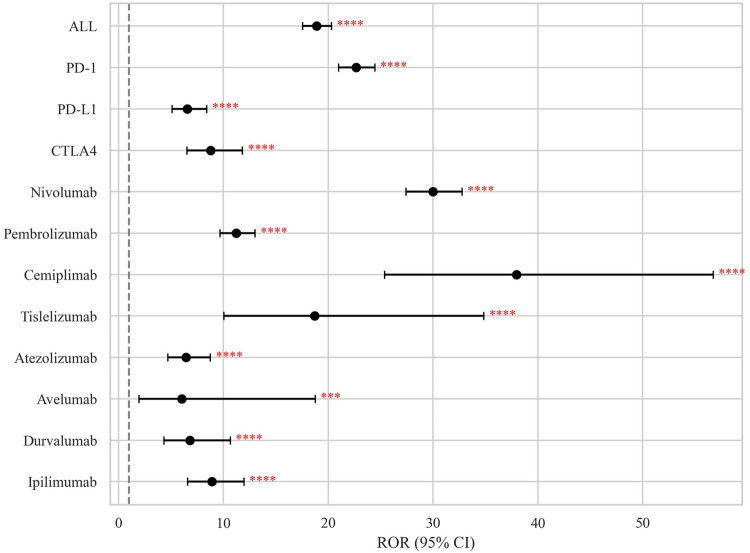
Disproportionality signals of ICIs related BP in the FAERS database. The dashed line indicates that ROR = 1.NS, no significance; *p < 0.05; **p < 0.01; ***p < 0.001; ****p < 0.0001.

At the individual agent level, cemiplimab demonstrated the strongest association (ROR = 37.96, 95% CI 25.40–56.73), followed by nivolumab (ROR = 29.99, 95% CI 27.43–32.78) and tislelizumab (ROR = 18.72, 95% CI 10.06–34.84) (Figure 3; Table 2).

**TABLE 2:** Disproportionality analysis of irBP.

Treatment	Number of cases	ROR (95% CI)	PRR (χ^2^)	MGPS(EBGM_05_)	BCPNN (IC_025_)
All ICIs	850	18.90 (17.58–20.31)	18.86 (12,425.30)	16.43 (15.29)	4.04 (3.9)
PD-1i	744	22.66 (20.99–24.47)	22.61 (13,542.03)	20.04 (18.56)	4.32 (4.2)
Nivolumab	530	29.99 (27.43–32.78)	29.90 (13,550.60)	27.45 (25.11)	4.78 (4.6)
Pembrolizumab	180	11.24 (9.69–13.04)	11.23 (1,629.07)	10.93 (9.43)	3.45 (3.2)
Cemiplimab	24	37.96 (25.40–56.73)	37.81 (856.81)	37.67 (25.21)	5.24 (3.4)
Tislelizumab	10	18.72 (10.06–34.84)	18.69 (167.16)	18.66 (10.03)	4.22 (2.0)
Dostarlimab	-	-	-	-	-
PD-L1i	62	6.55 (5.10–8.41)	6.54 (288.20)	6.49 (5.05)	2.70 (2.2)
Atezolizumab	40	6.43 (4.71–8.77)	6.42 (181.95)	6.39 (4.68)	2.68 (2.0)
Durvalumab	19	6.81 (4.34–10.68)	6.80 (93.80)	6.79 (4.33)	2.76 (1.7)
Avelumab	3	6.05 (1.95–18.78)	6.05 (12.64)	6.05 (1.95)	2.60 (−0.1)
CTLA-4i	44	8.79 (6.53–11.83)	8.78 (301.33)	8.73 (6.49)	3.13 (2.5)
Ipilimumab	44	8.79 (6.53–11.83)	8.78 (301.33)	8.73 (6.49)	3.13 (2.5)
Tremelimumab	-	-	-	-	-
Combination therapy					
Novi + Ipi	63	12.71 (9.92–16.30)	12.70 (672.19)	12.58 (9.81)	3.65 (3.1)
Ate + Ipi	4	24.14 (9.05–64.43)	24.08 (88.45)	24.07 (9.02)	4.59 (0.8)
Prem + Ipi(False)	2	14.80 (3.70–59.28)	14.78 (25.69)	14.78 (3.69)	3.89 (−0.3)
Dur + Tre(False)	1	2.48 (0.35–17.61)	2.48 (0.88)	2.48 (0.35)	1.31 (−1.5)
Ate + Tre(False)	2	35.96 (8.97–144.23)	35.82 (67.69)	35.81 (8.93)	5.16 (−0.2)

CTLA-4i, CTLA-4, inhibitor; PD-L1i, PD-L1, inhibitor; PD-1i, PD-1, inhibitor; ROR, reporting odds ratio (ROR >1, 95% CI >1, N ≥3); CI, confidence interval; PRR, proportional reporting ratio (PRR ≥2, χ^2^ ≥ 4, N ≥ 3); MGPS, multi-item gamma Poisson shrinker (EBGM_05_ >2); EBGM, empirical Bayesian geometric mean; EBGM_05_, lower limit of the one-sided 95% CI, of EBGM; BCPNN, Bayesian confidence propagation neural network (IC_025_ >0). “False” indicates that N < 3, without positive signal formation.

### Time-to-onset (TTO) analysis and temporal risk pattern analysis

Valid TTO data were available for 249 AE reports (29.29%). The median onset time to irBP was 165.2 days (IQR: 56–410). When stratified by ICI class, the median TTO differed significantly:

PD-1 inhibitor-related BP: 190.5 days, (IQR: 62–425)
PD-L1 inhibitor-related BP: 81 days, (IQR: 13.5–242.2)
CTLA-4 inhibitor-related BP: 35.7 days, (IQR: 9–84).

**FIGURE 4:**
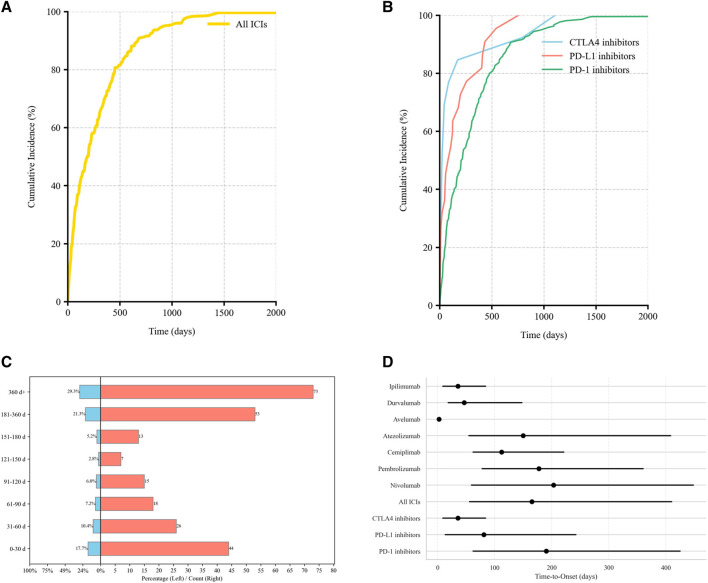
Time-to-onset (TTO) distribution of irBP. **(A)** The cumulative distribution curves for irBP. **(B)** The cumulative distribution curves for three ICIs. **(C)** Distribution of TTO. **(D)** The TTO for each drug.

The cumulative incidence curves showed that 17.7% of BP cases occurred within the first month of treatment, while 50.6% occurred after 6 months of therapy (Figure 4). Notably, PD-1 inhibitors demonstrated a significantly higher cumulative incidence rate over time compared to CTLA-4 inhibitors (adjusted *p* = 0.014; Table 3).

**TABLE 3:** Mann-Whitney U test for time-to-onset of ICIs-related BP.

Group 1	Group 2	U statistic	Raw *p*-value	Adjusted p-value (Bonferroni)	Significance
All ICIs	CTLA-4i	2350.5	0.006025	0.036148	Significant (*p* < 0.05)
All ICIs	PD-L1i	3,410.5	0.056873	0.34124	NS
All ICIs	PD-1i	25,239.5	0.328347	1	NS
CTLA-4i	PD-L1i	108	0.238675	1	NS
CTLA-4i	PD-1i	694	0.002449	0.014696	Significant (*p* < 0.05)
PD-L1i	PD-1i	1,647.5	0.020595	0.123572	NS

NS., not significant; CTLA-4i, CTLA-4, inhibitor; PD-L1i, PD-L1, inhibitor; PD-1i, PD-1, inhibitor.

To further characterize temporal risk pattern of BP onset, we applied the WSP model. All ICI categories demonstrated a shape parameter β < 1, indicating an early failure type where the risk of BP onset peaks shortly after treatment initiation and subsequently decreases.

Significant inter-class differences emerged:

CTLA-4 inhibitors showed a sharply concentrated early-onset risk window (β = 0.48, 95% CI: 0.41–0.91).
PD-L1 inhibitors exhibited intermediate risk concentration (β = 0.69, 95% CI: 0.56, 0.95)
PD-1 inhibitors displayed the broadest early-onset patterns (β = 0.83, 95% CI:0.72, 0.98) (Table 4).

**TABLE 4:** Weibull shape parameter test for ICIs-related BP.

Drug	Cases (n)	TTO (days)		Weibull shape parameter		Failure type
		Median (IQR)	Min–max	α (95% CI)	β (95% CI)	
PD-1i	214	190.5 (62–425)	1–7426	295.85 (252.96, 345.92)	0.83 (0.72, 0.98)	Early
PD-L1i	22	81 (13.5–242.2)	2–756	137.83 (69.24, 223.69)	0.69 (0.56, 0.95)	Early
CTLA-4i	13	35.7 (9–84)	1–1,108	76.24 (21.45, 195.16)	0.48 (0.41, 0.91)	Early
All ICIs	249	165.2 (56–410)	1–7426	264.3 (231.92, 313.6)	0.78 (0.69, 0.92)	Early

α = scale parameter; β = shape parameter. β < 1 indicates an early failure pattern.

The scale parameter α, representing the spread of TTO distribution, was highest with PD-1 inhibitor (α = 295.85), consistent with prolonged and variable onset. CTLA-4 inhibitors had the lowest α (76.24), supporting a tightly clustered onset pattern.

## Discussion

The increasing application of ICIs has significantly improved oncological outcomes, but various irAEs have also been reported. In particular, BP represents a rare but potentially serious cirAE, with this study identifying 51% mortality and 24% life-threatening outcomes among affected patients. Importantly, considering that ICIs are indicated for high mortality diseases, the primary cause of death and other detrimental outcomes may be attributed to disease progression rather than direct treatment toxicity.

In the current study, we provided a comprehensive pharmacovigilance analysis of irBP encompassing 850 documented cases. Consistent with previous findings [12], irBP occurred more commonly in males (67.8%) than females (22.0%). However, the global incidence rates of classical BP reveal a slightly higher rate in females (0.0202 per 1,000 person-years) compared to males (0.0181 per 1,000 person-years) [13]. This discrepancy may be attributed to the male predominance of certain types of cancer, such as melanoma, lung cancer, and renal cell carcinoma, which are major indications for ICIs [14–16]. The utilization patterns of ICIs in Korea also showed that the proportion of males (76.3%) was higher than that of females [17]. The most common age group was over 60 years (70.1%), which is consistent with the global incidence for different age groups [13]. Geographically, Japan accounted for the largest share of reports (29.4%), followed by the United States (26.7%) and France (16.2%). Notably, genetic polymorphism increases the risk of irBP [18], while ethnic differences play a role in genetic susceptibility to BP [19], which may also be the case in irBP.

Among irBP cases treated with ICIs, the majority occurred in patients treated for skin/melanoma (30.6%, mainly malignant melanoma), lung (22.6%, mainly non-small cell lung cancer), and kidney/renal malignancies (14.4%, mainly metastatic renal cell carcinoma), with smaller proportions in gastrointestinal, head and neck, bladder/urinary, and liver cancers. This distribution is consistent with prior epidemiological reports [3, 10, 12], confirming melanoma as the most prevalent underlying malignancy. Melanoma was associated with significantly increased odds of developing irBP after ICI treatment (adjusted OR = 3.21; 95% CI, 1.51–6.58) [10], potentially attributable to tumor-specific express of BP180 autoantigen triggering the production of anti-BP180 autoantibodies upon ICI-induced loss of immune tolerance [20]. While this mechanistically explains melanoma’s predisposition, the pathophysiological links between lung/renal cancer and BP remain unestablished, warranting further studies investigation.

Our disproportionality analysis detected significant BP signals across all four pharmacovigilance metrics (RORs, PRRs, BCPNN, and MGPS). PD-1 inhibitors consistently demonstrated the strongest class-level association with BP, exceeding signals from CTLA-4 and PD-L1 inhibitors across all methodologies. This result is concordant with previous pharmacovigilance studies about cirAEs [21]. At the agent level, cemiplimab (PD-1 inhibitor) monotherapy (ROR 37.96, PRR 37.81, EBGM_05_ 25.21, IC_025_ 3.4) and the combination of atezolizumab (PD-L1 inhibitor) with ipilimumab (CTLA-4 inhibitor, ROR 24.14, PRR 24.08, EBGM_05_ 24.07, IC_025_ 4.59) constitutes the most significant risks for irBP.

These findings corroborate previous FAERS-based analyses indicating a significant association between ICIs and BP, with PD-1 inhibitors generally showing elevated signal intensities (our ROR = 22.66; Tan et al. ROR = 24.45), supporting PD-1 blockade’s distinct role in BP pathogenesis. Methodologically, our study’s concurrent reporting of PRR, BCPNN, and MGPS allows for robust cross-algorithm validation of the ROR signals, reducing bias from reliance on a single method. Through agent-level stratification, cemiplimab and nivolumab are recognized as high-risk agents—an advancement beyond the class-level analysis by Tan et al.

Our analysis further identified a distinct hierarchy: PD-1 > CTLA-4 > PD-L1 inhibitors (ROR: 22.66 > 8.79 > 6.55). This contrasts with Tan et al.'s reported ranking (PD-1 > PD-L1 > CTLA-4) [12]. These discrepancies highlight the importance of methodological transparency in pharmacovigilance studies. Likewise, at the agent level, the extremely high-risk magnitudes demonstrated by cemiplimab (ROR = 37.96) and nivolumab (ROR = 29.99) demand the highest level of clinical vigilance.

TTO analysis indicated that the median onset time of irBP was 165.2 days (IQR 56–410), with 17.7% (44/249) of cases occurring within the first month and 50.6% (126/249) emerging after 6 months. This profile is generally consistent with the 204-day median (IQR 57–426) reported by Tan et al., [12] with minor differences possibly reflecting variations in observation periods (our inclusion of earlier cases from 2004 onward) and varying proportion of cases with valid TTO records.

To better understand the temporal dynamics of BP risk, we employed WSP modeling. All β values were <1, suggesting a declining hazard pattern—a characteristic of early-onset events. Among different ICI classes, CTLA-4 inhibitors exhibited the most marked early risk concentration (β = 0.48), whereas PD-L1 (β = 0.69) and PD-1 (β = 0.83) inhibitors exhibited a more extended risk period. Our quantitative confirmation of early failure patterns (β < 1) across all ICI classes complements Tan et al.’s clinical recommendation for long-term monitoring while emphasizing an early high-risk window, particularly for CTLA-4 blockade. Clinically, these findings highlight the importance of surveillance strategies stratified by risk magnitude. For instance, the rapid (median 35.7 days) and highly concentrated early-onset risk window for CTLA-4 inhibitors (β = 0.48), necessitates high-frequency dermatologic evaluation within the first month of initiating blockade. In contrast, PD-1 inhibitors not only carry the highest risk magnitude but also exhibit a much broader risk period (β = 0.83, median TTO 190.5 days), with 50.6% of cases emerging after 6 months. This risk magnitude profile compels the need for long-term, continued vigilance for patients on PD-1/PD-L1 therapies, extending well beyond the initial 6 months. Recognizing and leveraging the distinct “risk magnitude” and “temporal magnitude” across ICI classes and individual agents to design stratified surveillance strategies directly improves the timely detection and effective management of irBP, which is critical for optimizing clinical outcomes.

The limitations of this study inherent to pharmacovigilance databases. First, FAERS database has a voluntary nature with non-peer-reviewed AE data, potentially introducing unmeasured confounding. Second, a causal relationship cannot be established between ICIs and the onset of BP because of a disproportionality analysis. Third, absence of prescription denominator data precludes incidence calculation. Given these limitations, prospective studies are required to confirm these findings.

## Data Availability

The original contributions presented in the study are included in the article/Supplementary Material, further inquiries can be directed to the corresponding author.

## Footnotes

1
https://fis.fda.gov/

2
https://www.r-project.org/
