# Supplementary material for: Refined pharmacovigilance assessment of immune checkpoint inhibitors-related bullous pemphigoid: a multi-methodological approach utilizing FAERS database
Source: J Pharm Pharm Sci. 2026 Jan 7;28:15597. doi: 10.3389/jpps.2025.15597 (PMC12819333; doi:10.3389/jpps.2025.15597)
Supplement: Supplementary file 1 [file DataSheet1.pdf]

Supplement Table 1. Calculation formulas and threshold settings for the four adverse event signal detection methods

methods	Calculation formulas	threshold
ROR	$\mathrm{ROR} = \frac{a \cdot d}{b \cdot c}$	ROR >1 & $low\_95CI > 1$ & $a \geq 3$
	$\mathrm{SE}_{\ln(\mathrm{ROR})} = \sqrt{\frac{1}{a} + \frac{1}{b} + \frac{1}{c} + \frac{1}{d}}$	
	$95\%CI = e^{\ln(\mathrm{ROR}) \pm 1.96 \cdot \mathrm{SE}_{\ln(\mathrm{ROR})}}$	
PRR	$PRR = \frac{\frac{a}{a+b}}{\frac{c}{c+d}}$	$PRR \geq 2$ & $x^2 \ \geq \ 4$ & $a \geq 3$
	$\chi^2 = \frac{(a \cdot d - b \cdot c)^2 \cdot n}{(a+b)(a+c)(b+d)(c+d)}$	
BCPNN	$IC = \log_2 \frac{a \cdot n}{(a+b)(a+c)}$	$\mathrm{IC}_{025} > 0$
	$E(IC) = log_2 \frac{(a+\gamma 11)(a+b+c+d+\alpha)(a+b+c+d+\beta}{(a+b+c+d+\gamma)(a+b+\alpha 1)(a+c+\beta 1)}$	
	$V(IC) = \frac{1}{(l\,n\,2)^2}\left(\frac{(a+b+c+d)-a+\gamma-\gamma_{11}}{(a+\gamma_{11})(1+a+b+c+d+\gamma)} + \frac{(a+b+c+d)-(a+b)+\alpha-\alpha_1}{(a+b+\alpha_1)(1+a+b+c+d+\alpha)} + \frac{(a+b+c+d)-(a+c)+\beta-\beta_1}{(a+c+\beta_1)(1+a+b+c+d+\beta)}\right)$	
	$\gamma = \gamma_{11} \frac{(a+b+c+d+\alpha)(a+b+c+d+\beta)}{(a+b+a1)(a+c+\beta 1)}$	
	$IC_{025} = IC - 2SD = E(IC) - 2\sqrt{V(IC)}$	
$MGPS(EBGM)$	$EBGM = \frac{a \cdot n}{(a+c)(a+b)}$	$\mathrm{EBGM}_{05} > 2$
	$SE_{\ln(\mathrm{EBGM})} = \sqrt{\frac{1}{a} + \frac{1}{b} + \frac{1}{c} + \frac{1}{d}}$	
	$EBGM_{05} = e^{\ln(\mathrm{EBGM}) - 1.96 \cdot \mathrm{SE}_{\ln(\mathrm{EBGM})}}$	
